# Supplementary material for: A single-cell multi-omic atlas spanning the adult rhesus macaque brain
Source: Sci Adv. 2023 Oct 12;9(41):eadh1914. doi: 10.1126/sciadv.adh1914 (PMC10569716; doi:10.1126/sciadv.adh1914)
Supplement: Supplementary file 1 — Figs. S1 to S19 Legends for tables S1 to S19 [file sciadv.adh1914_sm.pdf]

Science Advances
AAAS

Supplementary Materials for

A single-cell multi-omic atlas spanning the adult rhesus macaque brain

Kenneth L. Chiou *et al.*

Corresponding author: Noah Snyder-Mackler, nsnyderm@asu.edu; Jay Shendure, shendure@uw.edu; Michael L. Platt, mplatt@pennmedicine.upenn.edu

Sci. Adv. **9**, eadh1914 (2023)
DOI: 10.1126/sciadv.adh1914

The PDF file includes:

Figs. S1 to S19
Legends for tables S1 to S19

Other Supplementary Material for this manuscript includes the following:

Tables S1 to S19
List of consortium authors

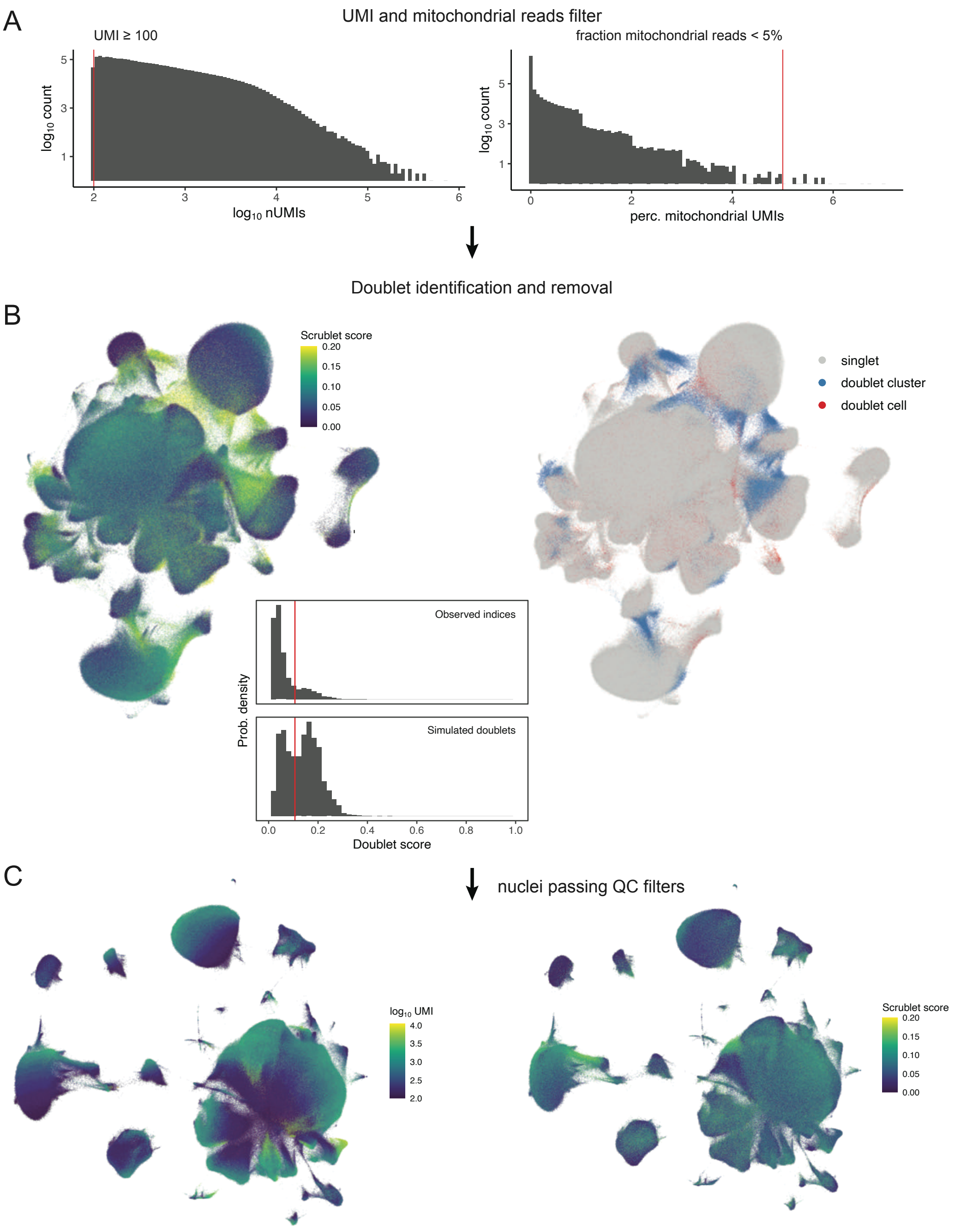

Figure S1. Schematic depicting snRNA-seq quality control pipeline. **A**, Nuclei (combinatorial indices) with fewer than 100 UMIs and greater than 5% reads mapping to the mitochondrial genome were removed. **B**, Scrublet *k*-nearest-neighbor (kNN) doublet scores were calculated per-sample and doublets with scores > 0.20 were marked (using automated Scrublet thresholds with manual adjustment) but not removed. All nuclei, including doublets, were then jointly preprocessed and clustered. Clusters with mean doublet scores > 0.15 were then removed along with previously marked doublets. **C**, UMI counts and Scrublet doublet-detection scores visualized on the post-quality-control dataset.

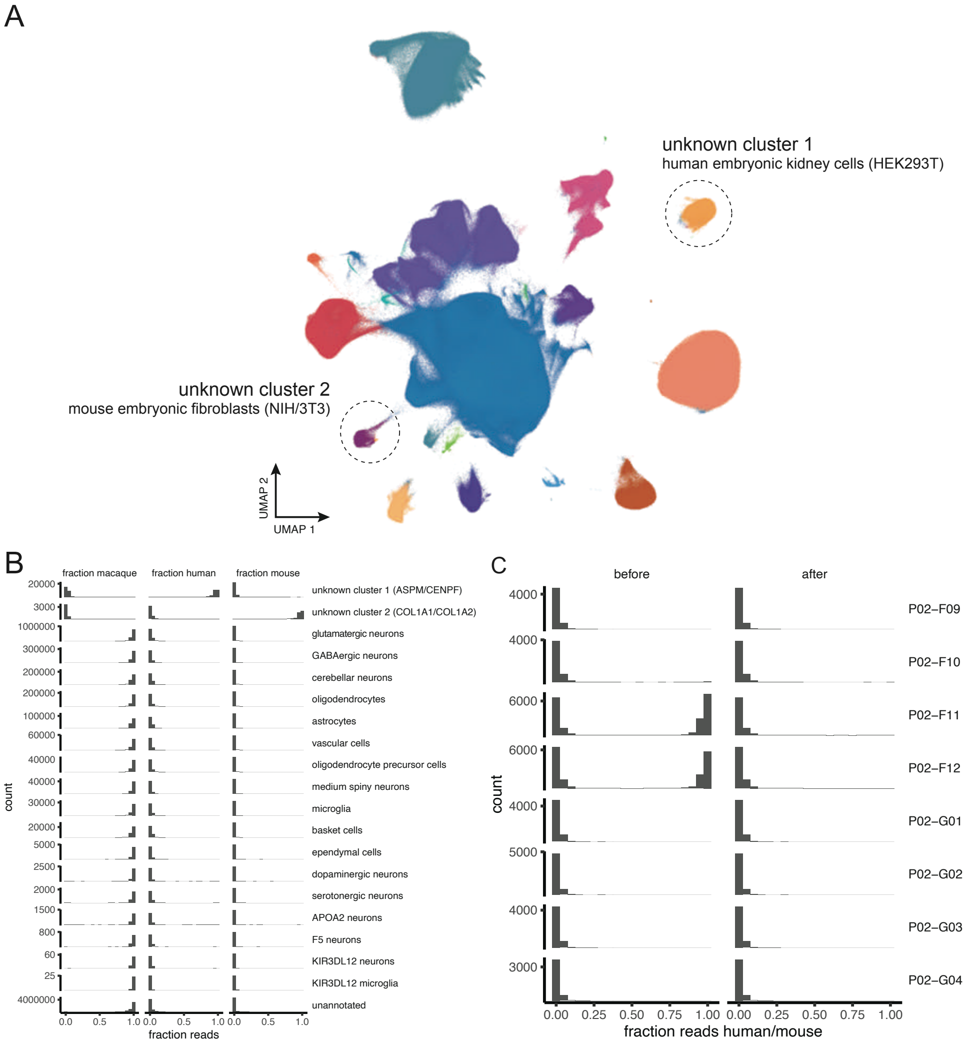

Figure S2. Identification and removal of exogenous nuclei. **A**, Two anomalous clusters were identified during the course of cell-type annotation had marker gene profiles (unknown cluster 1: *ASPM*, *CENPE*, *CENPF*, *MKI67*; unknown cluster 2: *COL1A1*, *COL1A2*, *FN1*, *VIM*) characteristic of stem cell progenitors. **B**, Using the BBSplit multi-genome mapping strategy, reads were assigned to either the rhesus macaque, human, or mouse genomes. Histograms showed that exogenous (human or mouse) reads were specific to the two anomalous clusters and identified them as human-derived (unknown cluster 1) and mouse-derived (unknown cluster 2), respectively. **C**, Histograms of exogenous read fractions reveal that exogenous reads were specific to particular reverse-transcription (RT) barcodes. The 8 barcodes shown (named according to plate number and position in 96-well plate) were assigned to equal aliquots of a single tissue sample, the right SPP from individual 2C0 (library NSM345). Reads associated with two barcodes (P02-F11 and P02-F12) showed clear evidence of contamination (notably, a human-mouse barnyard control sample was loaded in adjacent wells P02-G11 and P02-G12). After the two anomalous clusters were removed from the entire dataset, these two barcodes no longer showed discernible evidence of exogenous contamination, indicating that human- and mouse-derived nuclei had been effectively partitioned and removed from the dataset. We observed similar patterns with some other samples, though with much lower degrees of contamination.

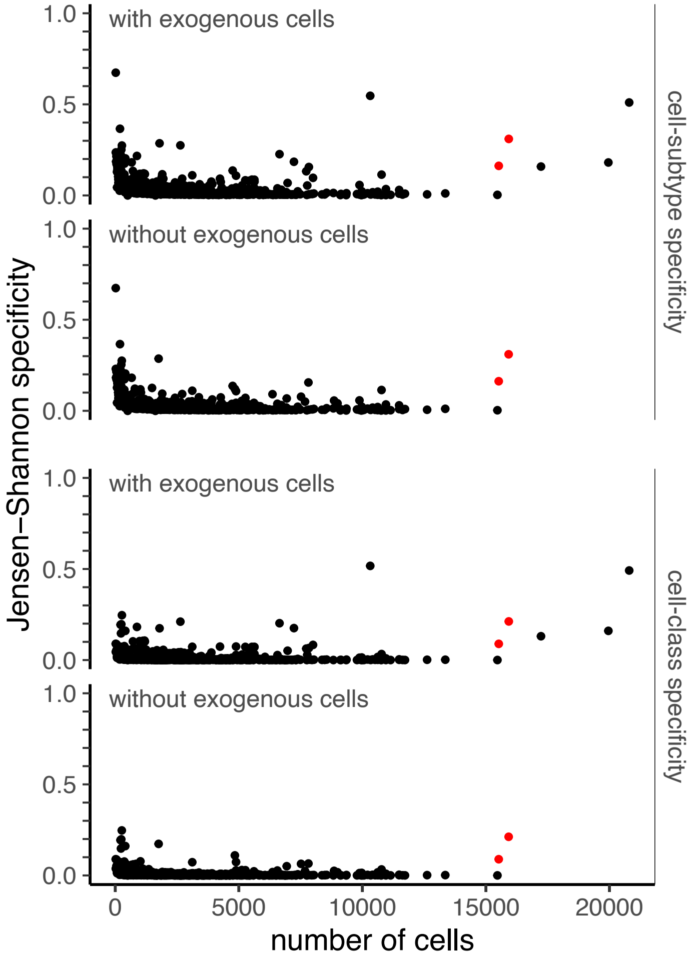

Figure S3. Identification of residual contamination. Our discovery of exogenous (non-macaque) cells (see **fig. S2**) called into question whether other instances of cross contamination, particularly contamination involving other macaque samples, could be detected. As each sample was run with multiple reverse-transcription wells/barcodes (typically at least 4), we scanned cell-subtype (top) and cell-class (bottom) proportions partitioned by barcode (and experiment) in order to identify barcodes with elevated Jensen-Shannon divergence, indicating diverged cell proportions from the sample-wide mean. Results are shown before and after the removal of exogenous cells. After excluding exogenous cells, we observed two barcodes with both a large number of cells and high specificity (indicated in red). We excluded all cells with these barcodes from downstream analyses of regional specificity and enrichment.

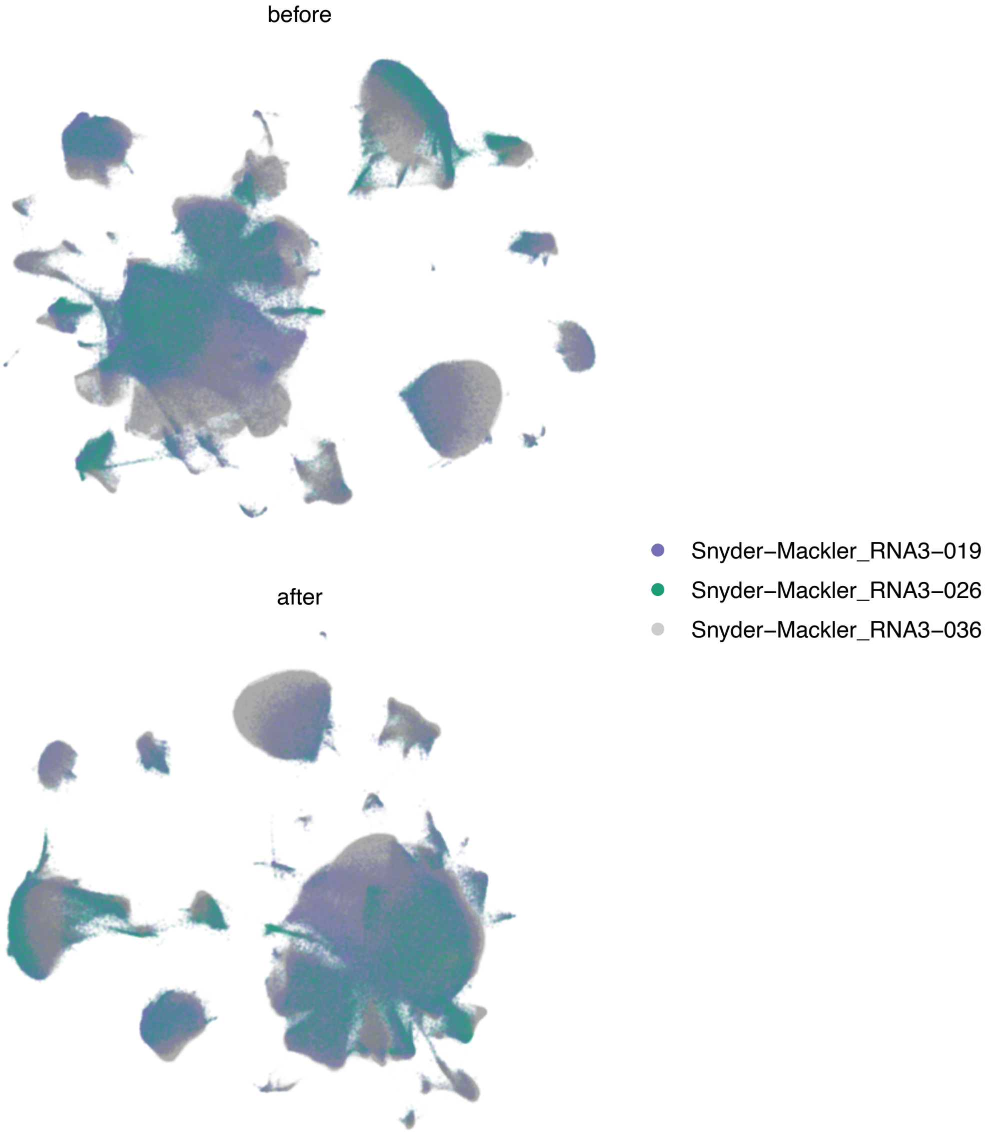

Figure S4. Comparison of UMAP projections before batch and after batch correction. The UMAP projection prior to batch correction was generated using the Scanpy ‘neighbors‘ function to build a neighborhood-graph while the UMAP projection with batch correction used BBKNN in place of ‘neighbors‘. Colors highlight the three library-preparation/sequencing batches, with the third batch shown in a lighter gray color with increased transparency due to the higher nuclei numbers from this batch implementing protocol improvements.

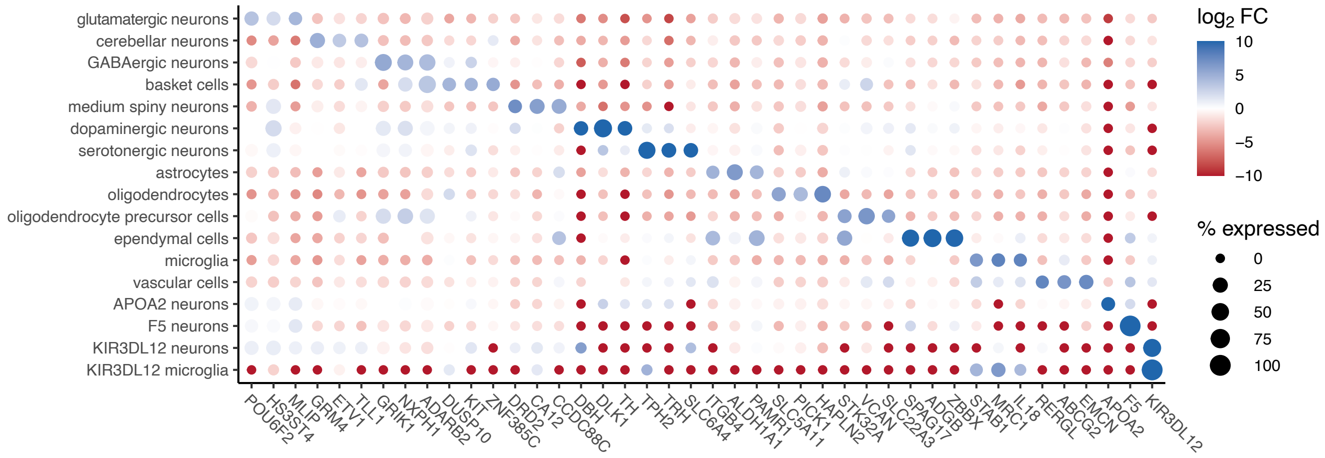

Figure S5. Dot plot showing the top three marker genes (ranked by regularized logistic regression coefficient) for each cell class identified by our analysis. Marker genes with percent expression < 10% were excluded, as were novel genes lacking gene names. For the four rarest cell classes, only the top marker is shown (*KIR3DL1/2* was the top marker gene for two rare cell classes). Log_2 fold-change (FC) color ranges are capped at ±10.

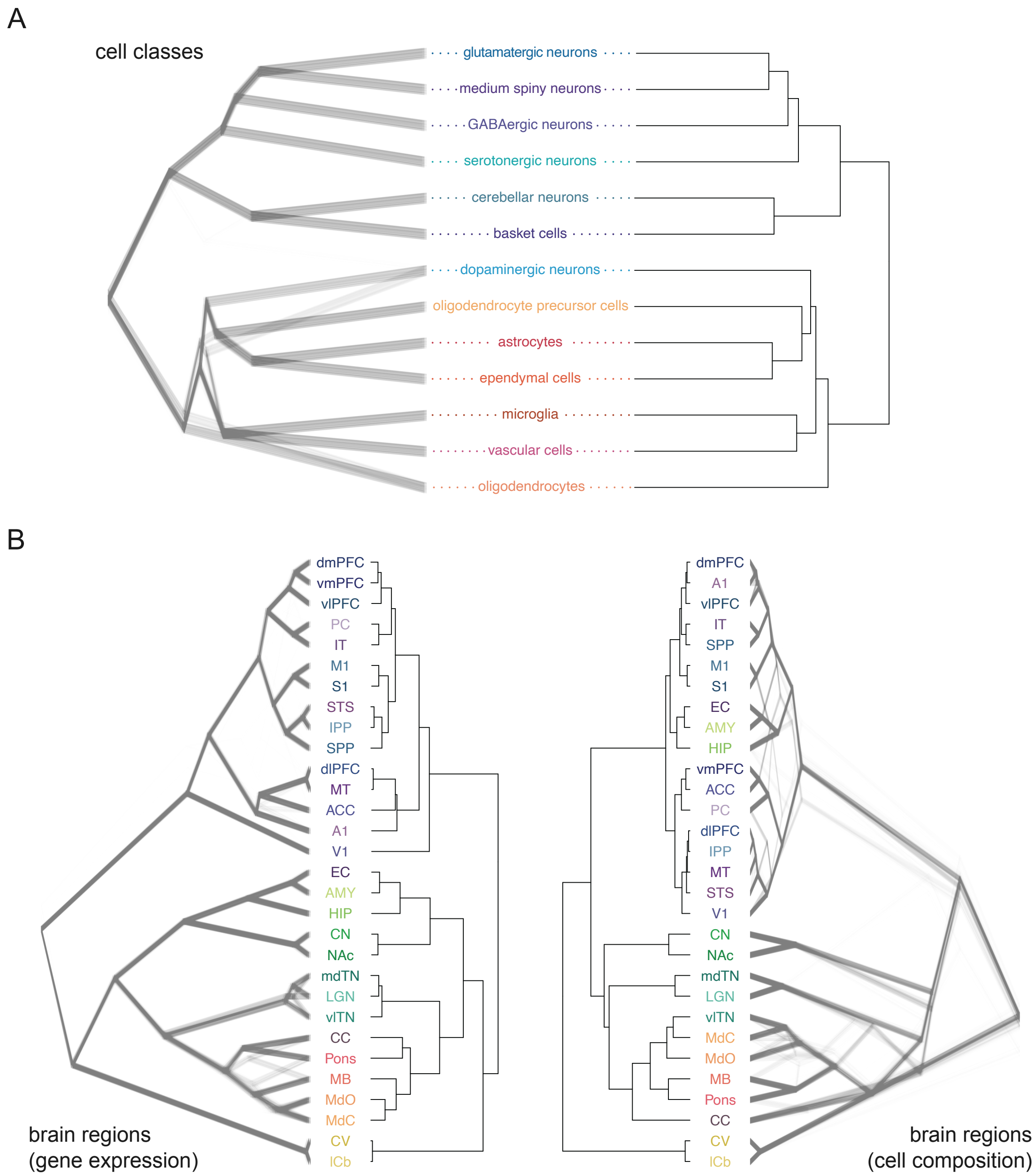

Figure S6. Unsupervised clustering of cell classes and brain regions. **A**, Dendrograms showing unsupervised hierarchical clustering of cell classes by the top 50 principal components of gene expression. The consensus tree is shown on the right, opposite an uncertainty tree derived from 1,000 bootstrap replicates. **B**, Dendrograms showing unsupervised clustering of brain regions by, left, the top 50 principal components of gene expression and, right, relative proportions of cell classes. Consensus trees are shown opposite uncertainty trees which were also each derived from 1,000 bootstrap replicates.

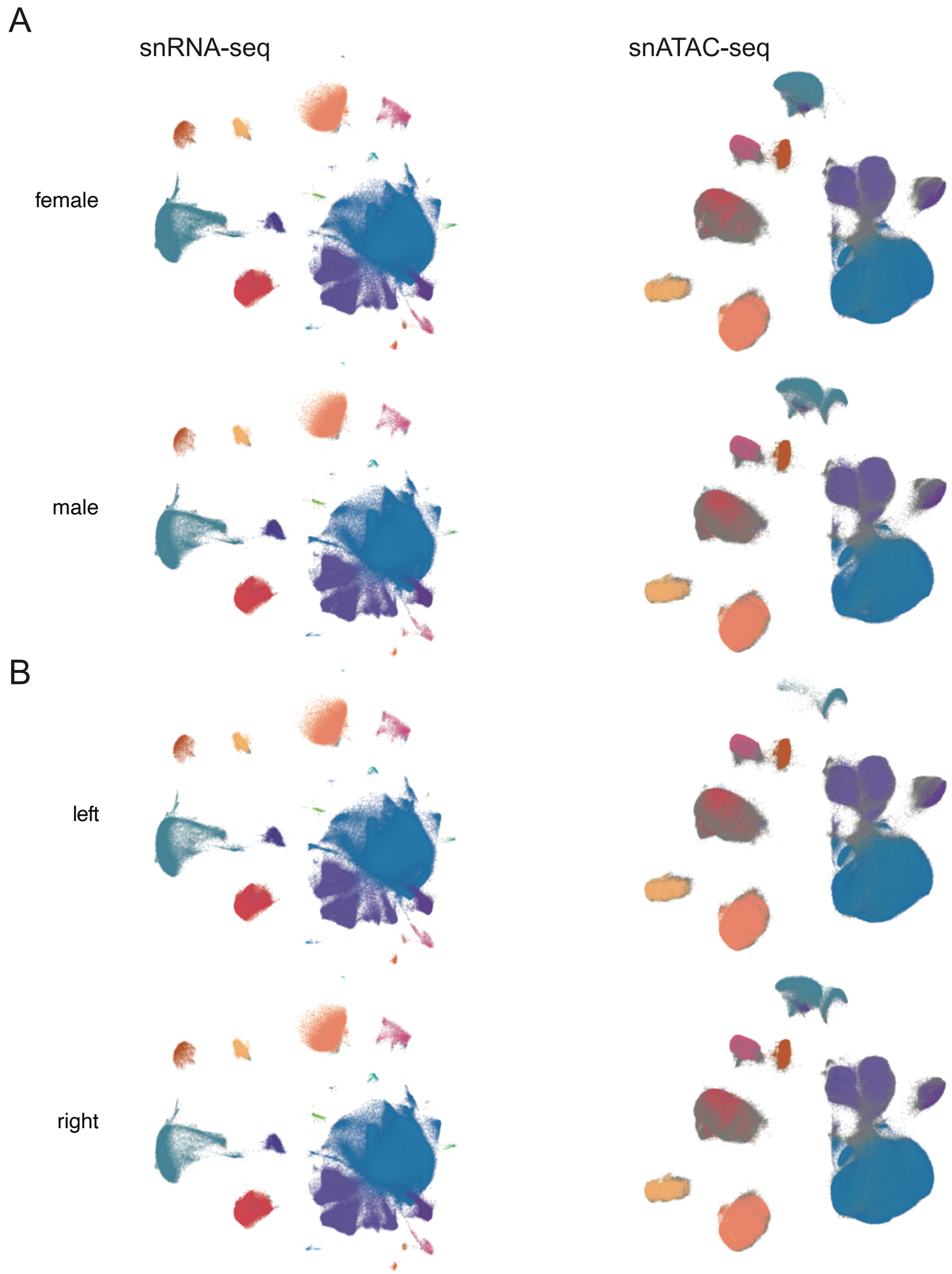

Figure S7. Sex and hemisphere balance. Comparison of UMAP embeddings of nuclei derived from samples of different **A**, biological sex and **B**, brain hemisphere. snRNA-seq data are shown on the left and snATAC-seq data are shown on the right. For the snATAC-seq dataset, nuclei lacking cell-class assignments are shown in gray. All other colors follow the color scale in **Fig. 1B** and **Fig. 2G**. For hemisphere comparisons, nuclei from the cerebellar vermis (Vrm) and midbrain (MB) are not shown because the structures are located on the midline and were sampled from either or both hemisphere(s) depending on where they were located.

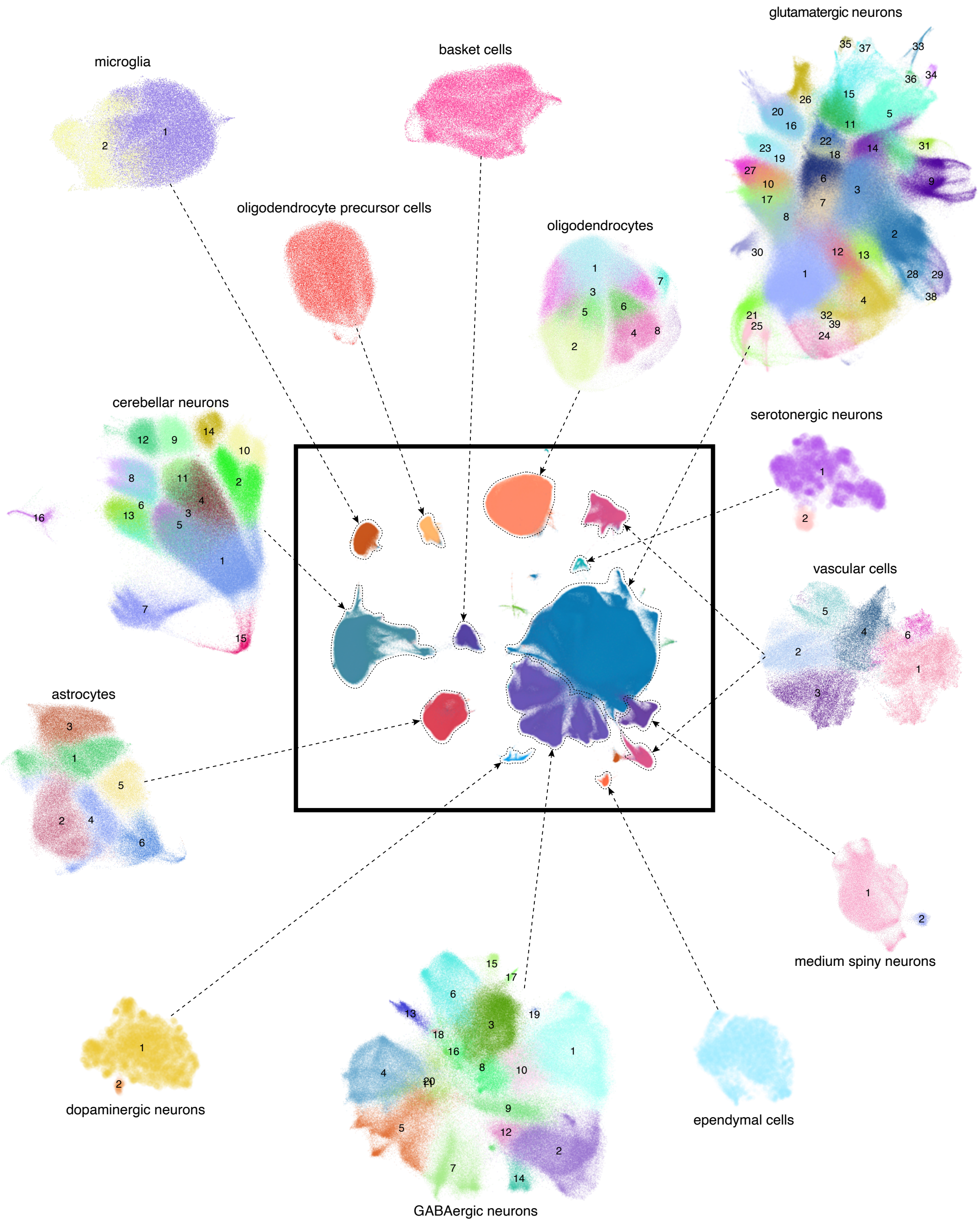

Figure S8. Cell-class specific UMAP projections colored and labeled according to identified cell subtypes. To identify cell subtypes, the dataset was partitioned by cell class and preprocessing, clustering, and annotation steps were repeated on each partition separately. Cell subtype colors were generated separately for each cell class partition using the randomcoloR/v.1.1.0.1 package in R.

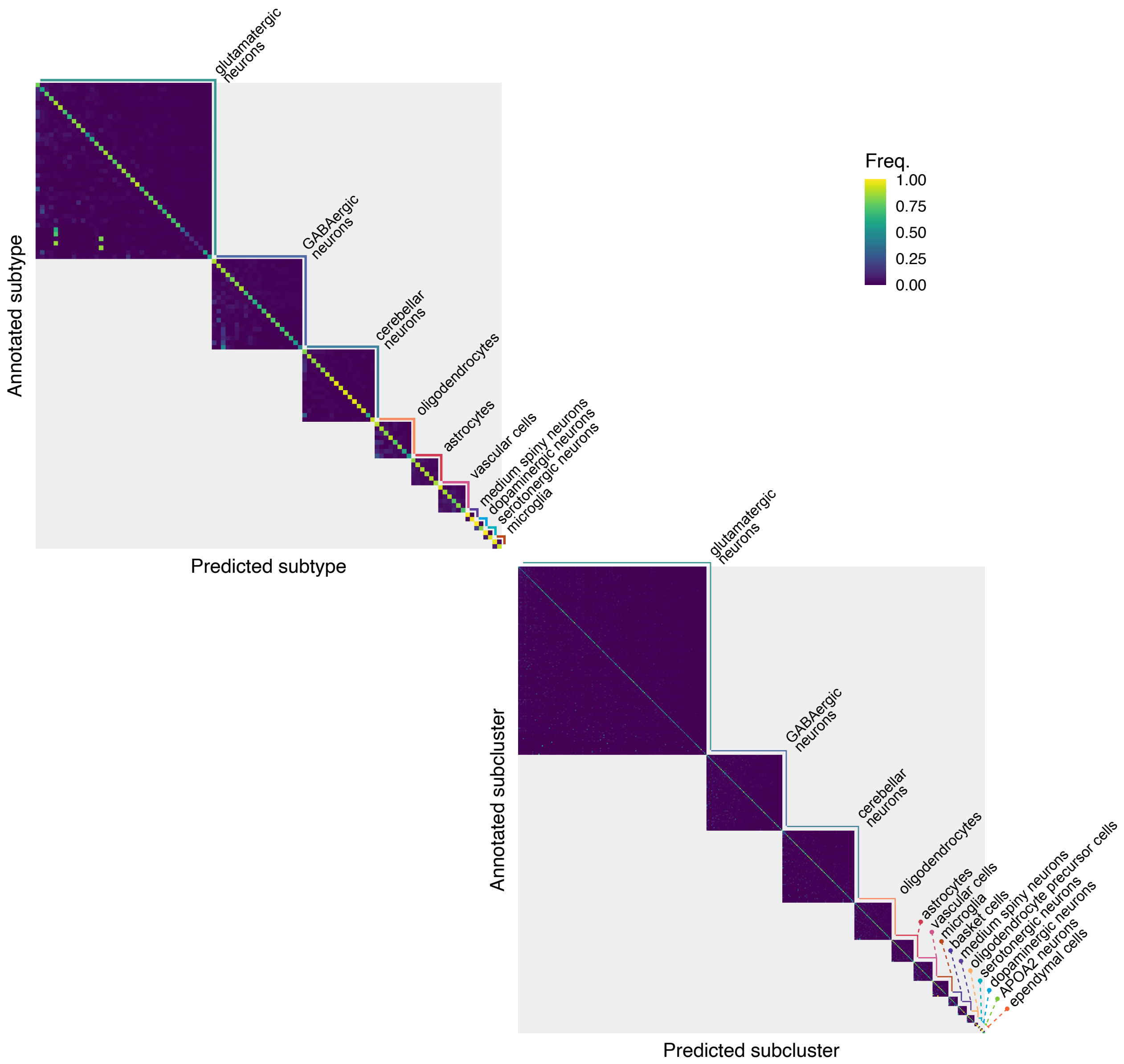

Figure S9. Cluster quality scores demonstrate robustness of cell subtype (top left) and cell subcluster annotations (bottom right). We used a support vector machine (SVM) classifier on normalized, log-transformed, and scaled gene expression data to predict cell annotations with 5-fold cross validation. The heatmaps here show the confusion matrix comparing true annotations (rows) and predicted annotations (columns), normalized such that the values in a given row add up to one. The presence of a clear diagonal demonstrates that cell-subtype and cell-subcluster annotations are robustly predicted by the classifier. We provide the accuracy values for each cell-subtype and cell-subcluster annotation in **table S7** and **table S8**, respectively.

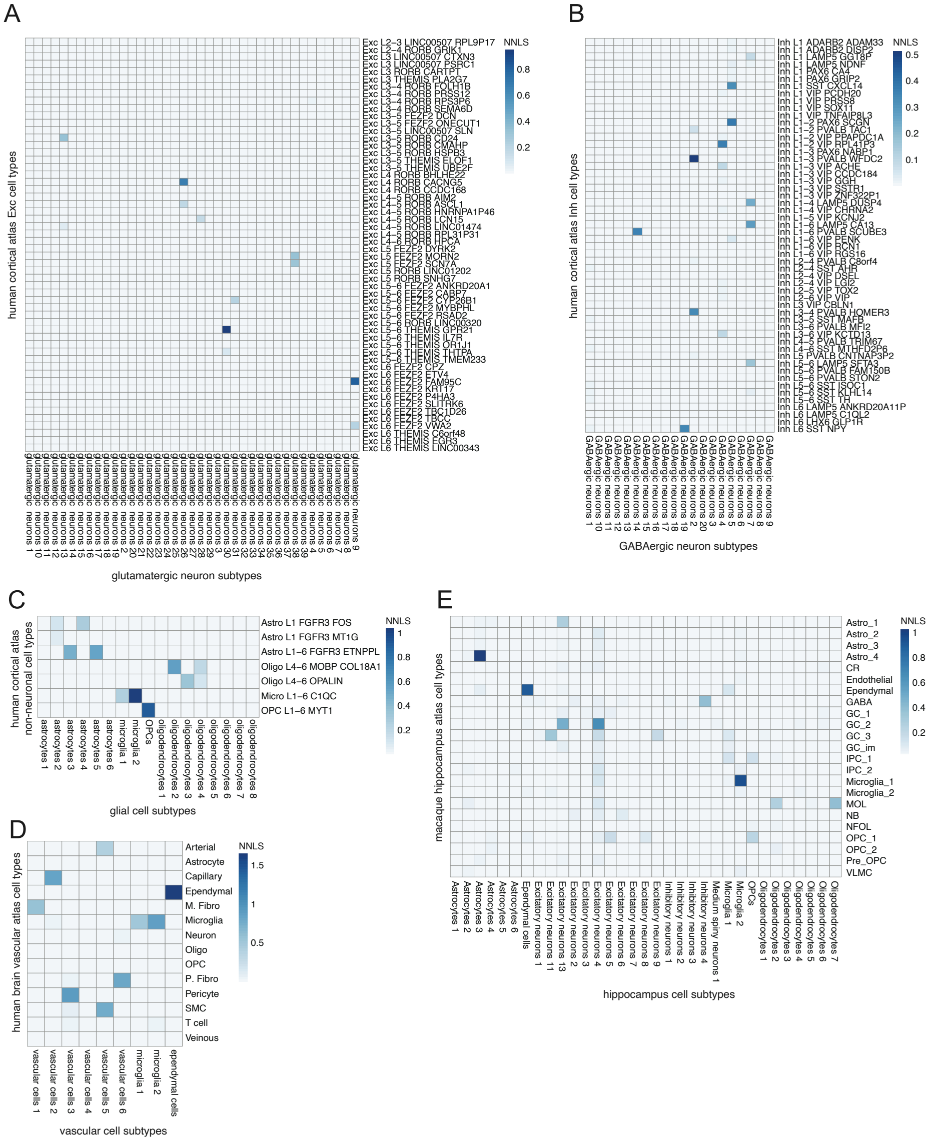

Figure S10. Cell subtype concordance with human cortical, human brain vascular cell and macaque hippocampus atlases. **A**-**C**, Correlations between cell subtypes and annotated labels in a cortical human brain atlas. Combined β values from bi-directional non-negative least squares (NNLS) regression are shown for adult macaque cell subtypes (x axis) and cortical cell types annotated in the Allen human cortex dataset comprising several cortical brain regions (y axis). Glutamatergic neuron, GABAergic neuron and glial subtypes are shown in three separate panels. **D**, Correlations between cell subtypes and annotated labels in a human brain vascular cell atlas. Combined β values from bi-directional NNLS regression are shown for adult macaque cell subtypes (vascular, myeloid, and ependymal cells, x axis) and reference cell types in the vascular cell atlas (y axis) . **E**, Correlations between cell subtypes and annotated labels in a macaque hippocampus cell atlas. Combined β values from bi-directional NNLS regression are shown for adult macaque glutamatergic neuron, GABAergic neuron and glial cell subtypes that are sufficiently abundant in the hippocampus (N>100, x axis) and reference cell types in the macaque hippocampus cell atlas (y axis).

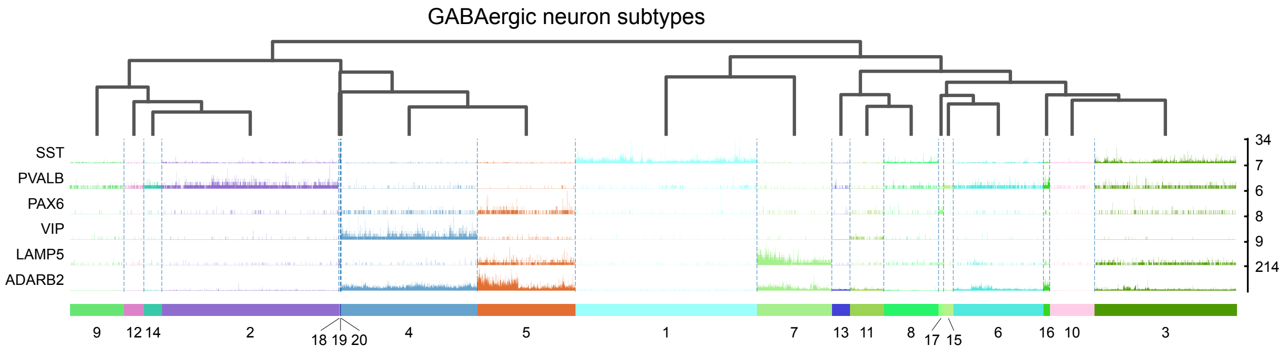

Figure S11. "Tracks plot" showing snRNA-seq read counts for cells assigned to each of 20 GABAergic neuron subtypes for six known GABAergic neuron markers. Dendrogram is based on hierarchical clustering of the top 50 principal components of gene expression in the GABAergic neuron class dataset partition.

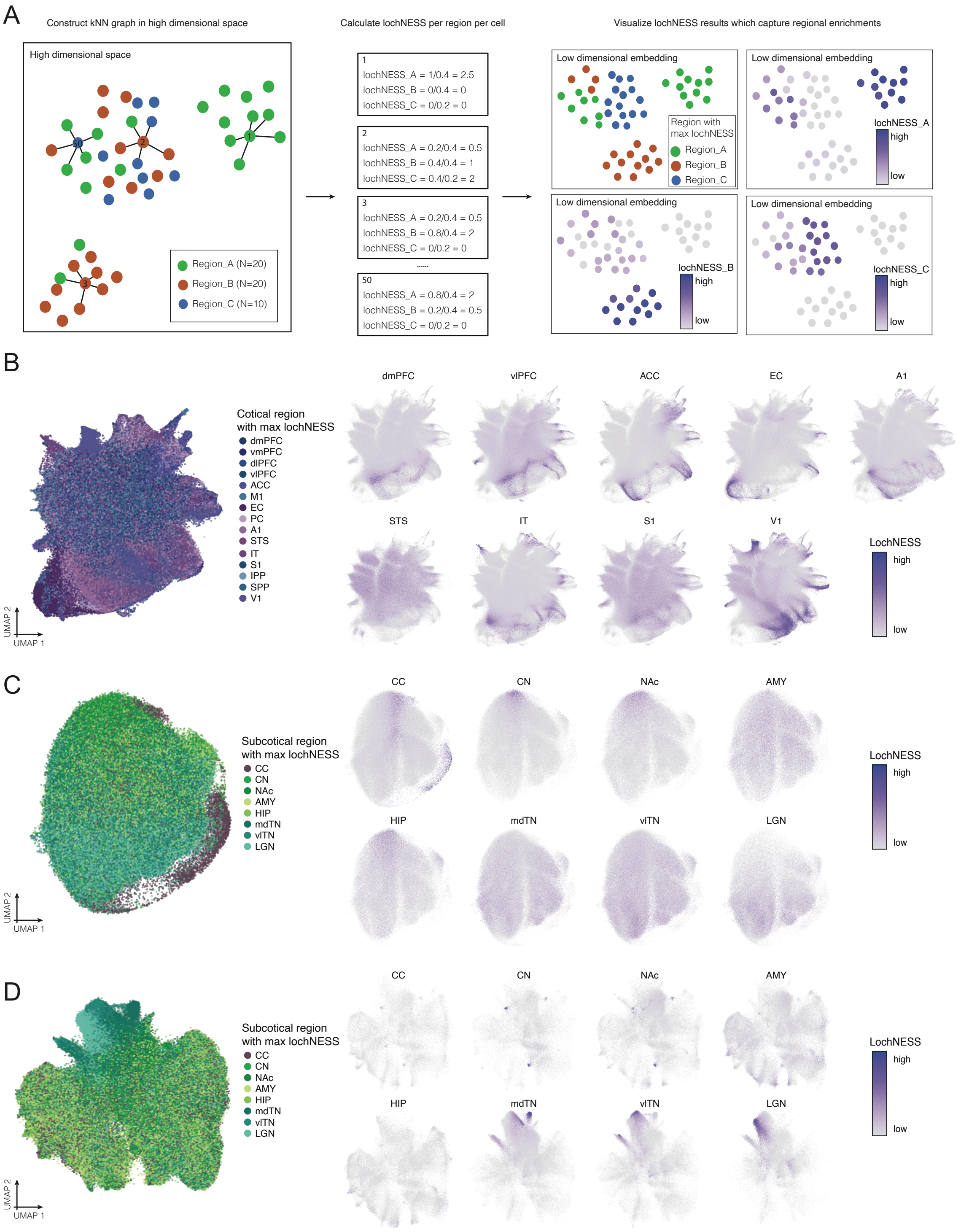

Figure S12. Schematic of extended lochNESS and additional lochNESS examples. **A**, Schematic of the lochNESS analysis. **B**, UMAP visualizations of glutamatergic neurons colored by the cortical region with the highest lochNESS (left). LochNESS distributions in a subset of cortical regions are shown in separate panels (right). **C**, UMAP visualizations of oligodendrocytes colored by the subcortical region with the highest lochNESS (left). LochNESS distribution in subcortical regions are shown in separate panels (right). **D**, UMAP visualizations of GABAergic neurons colored by the subcortical region with the highest lochNESS (left). LochNESS distribution in subcortical regions are shown in separate panels (right).

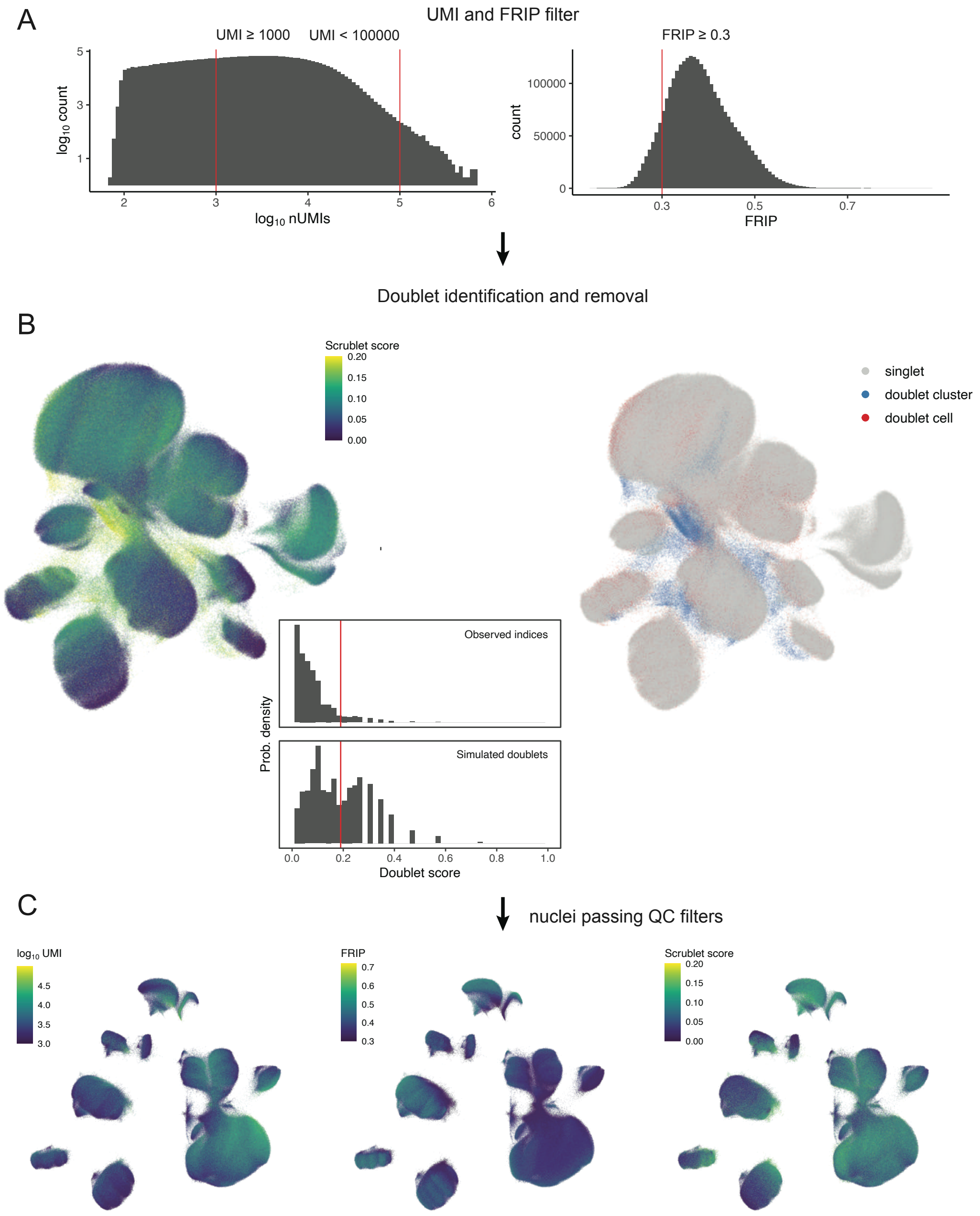

Figure S13. Schematic depicting snATAC-seq quality control pipeline. **A**, Nuclei (combinatorial indices) with fewer than 100 or greater than 100,000 UMIs were removed, as were nuclei with fractions of reads in peaks (FRIP) < 0.3. **B**, Scrublet *k*-nearest-neighbor (kNN) doublet scores were calculated per-sample and doublets with scores > 0.20 were marked (using automated Scrublet thresholds with manual adjustment) but not removed. All nuclei, including doublets, were then jointly preprocessed and clustered. Clusters with mean doublet scores > 0.15 were then removed along with previously marked doublets. **C**, UMI counts, FRIP, and Scrublet doublet-detection scores visualized on the post-quality-control dataset.

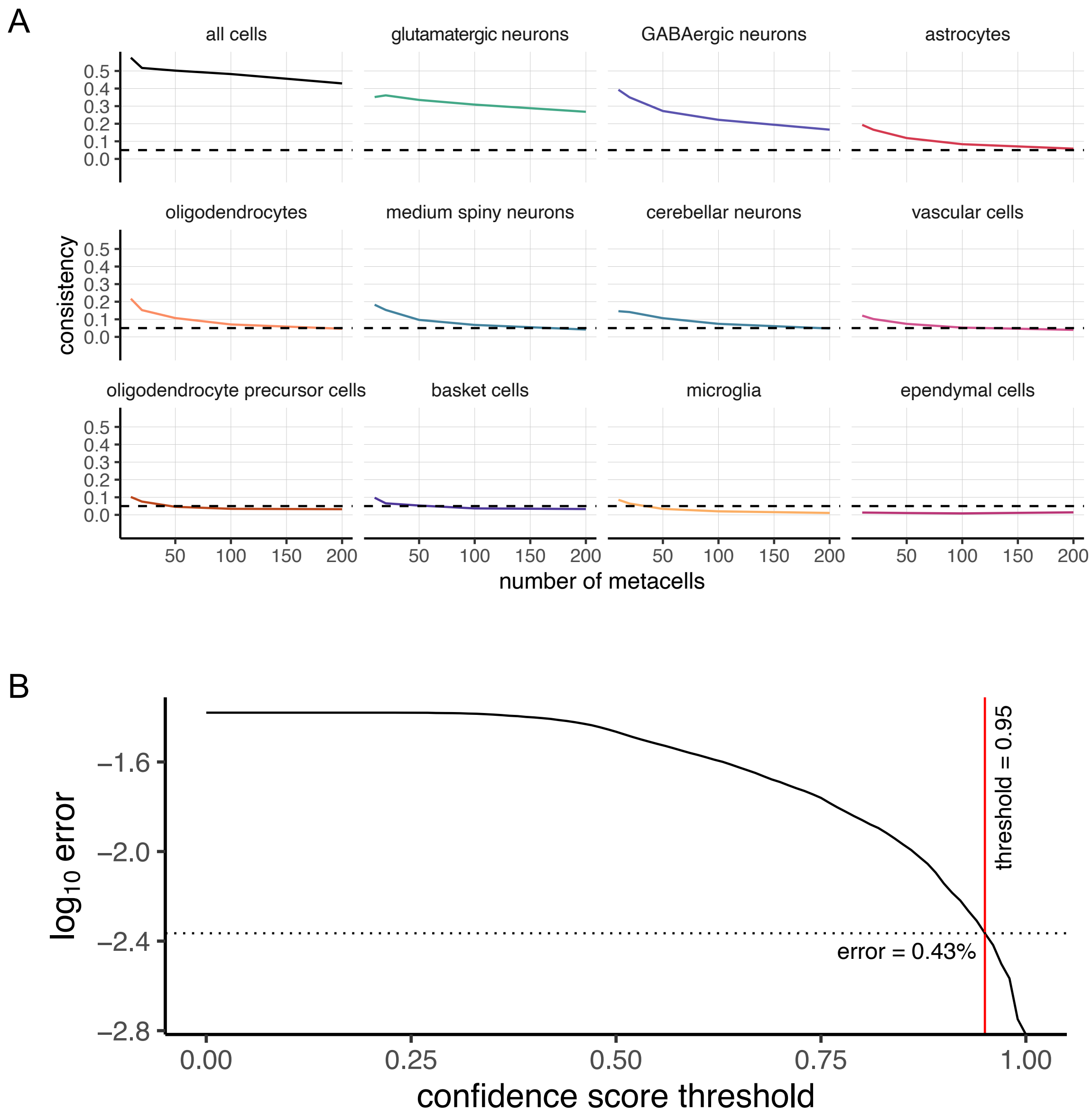

Figure S14. Assessment of snRNA-seq/snATAC-seq integration quality. **A**, Integration consistency scores—calculated by grouping neighboring cells into "metacells" and computing correlations—were calculated using glue and are plotted here. These plots include both integrations performed at the dataset-wide level ("all cells") and at the cell-class-specific level. Integrations are considered more reliable the higher the curve is. We observed that larger cell classes tended to have higher integration consistency scores. **B**, Cell-class prediction accuracy was calculated using an evaluation dataset of 100,000 snRNA-seq cells not used in the reference dataset. A range of confidence score thresholds were then tested. At a confidence threshold of 0.95 (the chosen threshold), the prediction error (percentage of incorrectly predicted cell-class labels) was 0.43%.

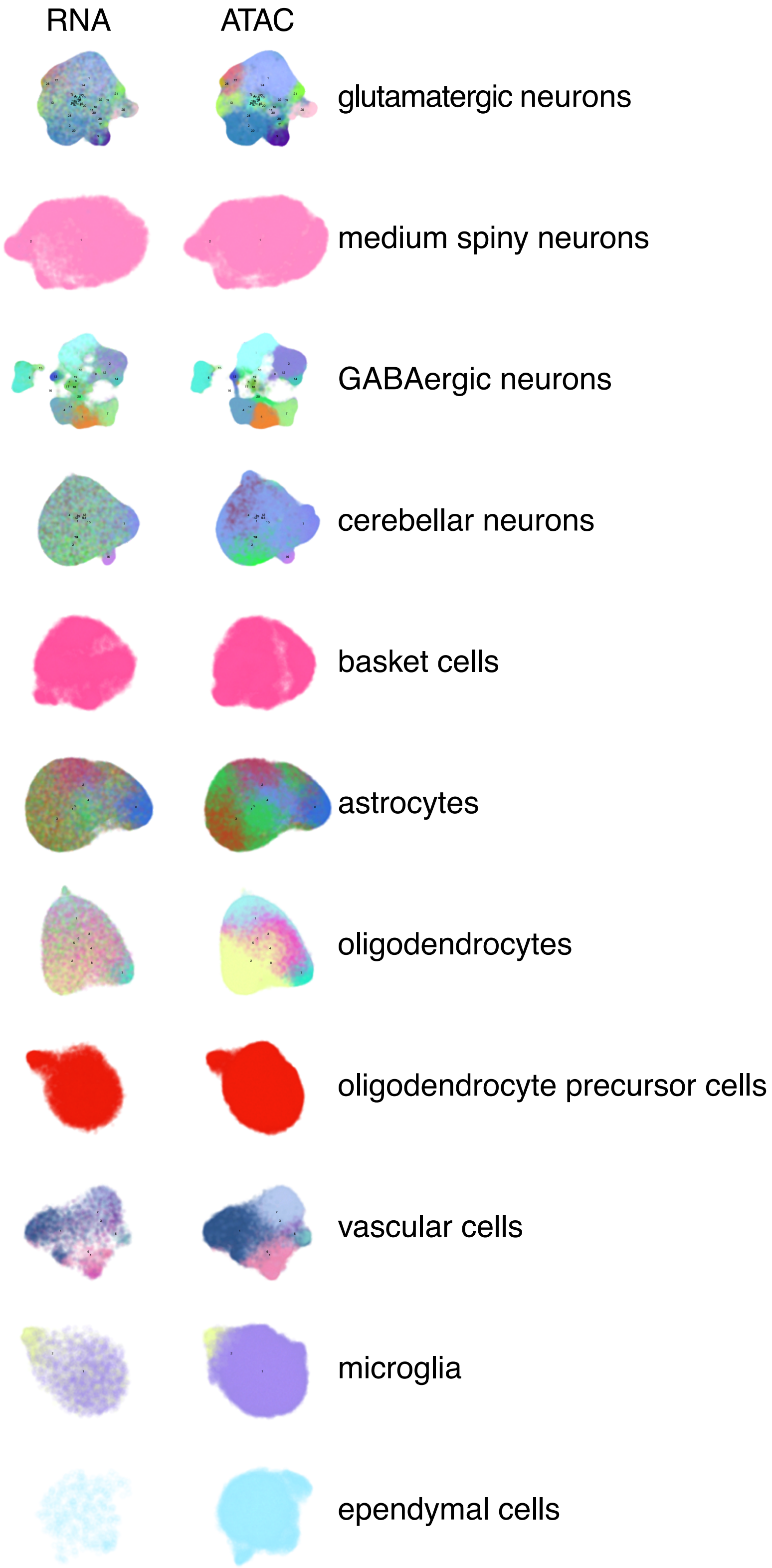

Figure S15. UMAP embeddings of snRNA-seq and snATAC-seq data integrated separately across cell classes. Cells are colored according to annotated or predicted cell subtypes and match the colors in **fig. S8**.

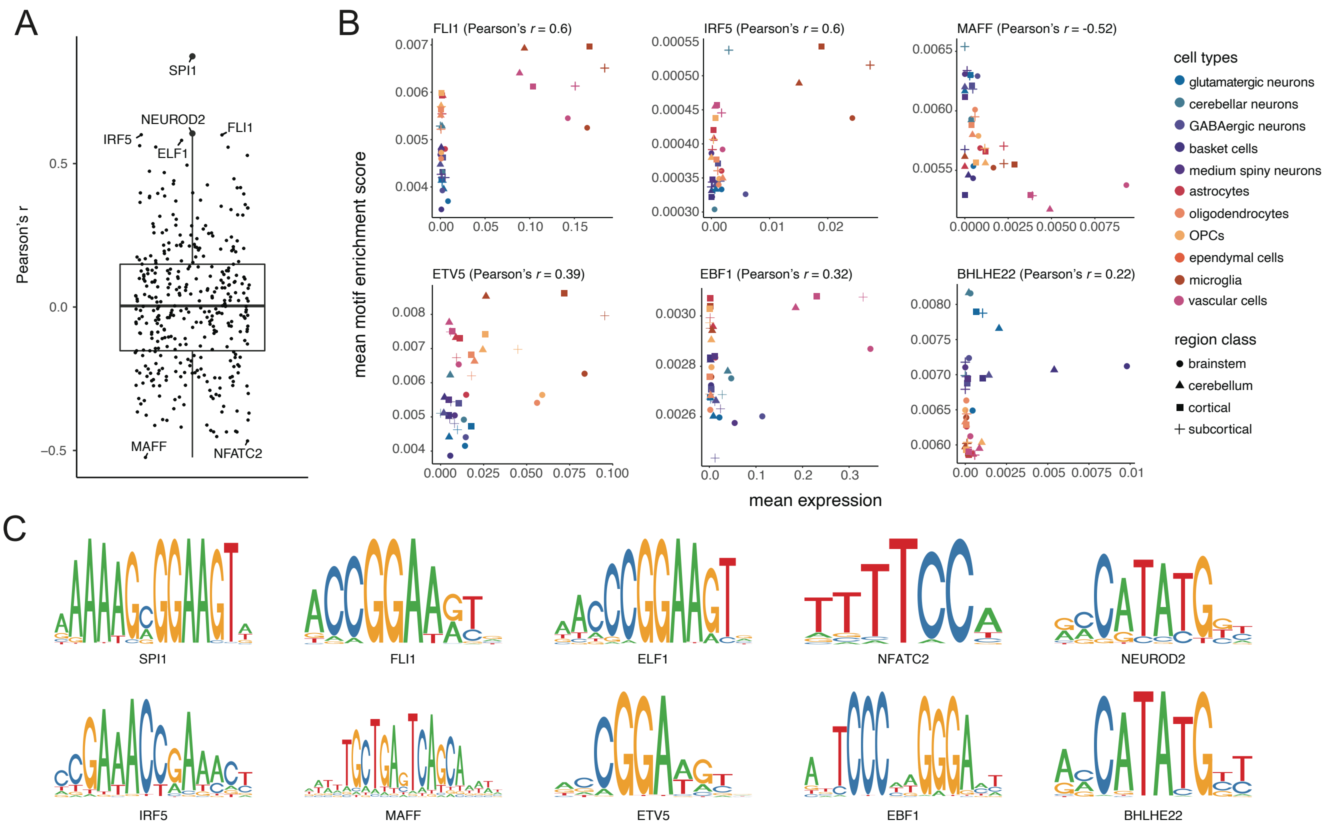

Figure S16. Correlation of TF expression and motif enrichments. **A**, Boxplot showing distribution of Pearson's correlation coefficients with TFs with largest coefficient values labeled. **B**, Scatterplots showing correlation between snATAC-seq accessibility of TF binding motifs and snRNA-seq gene expression of corresponding TF genes within cell classes in regional classes for six TFs (in addition to examples in **Fig. 4D**). These TFs with cell-class-specific activating and repressing effects were selected either by systematically screening for large coefficient values (top row) or manual inspection (bottom row). **C**, Position weight matrices of the ten TF motifs shown in the TF expression and motif enrichment correlation analysis.

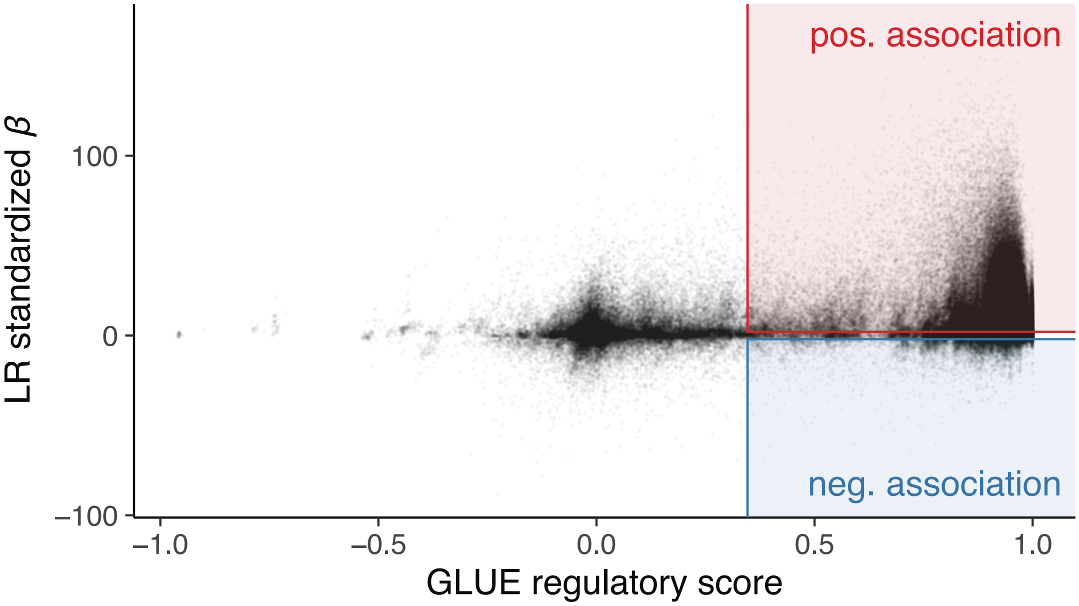

Figure S17. Concordance between GLUE regulatory scores—which measure the cosine similarity between genes and peaks in the integrated multidimensional embedding—and logistic regression standardized effect sizes (standardized β), calculated based on metacells. Shaded areas encompass peak-gene pairs that are identified as candidate regulatory interactions ($P_{adj} <$ 0.05 for both statistics), with the color depicting the direction of the association based on the sign of the logistic regression β estimate.

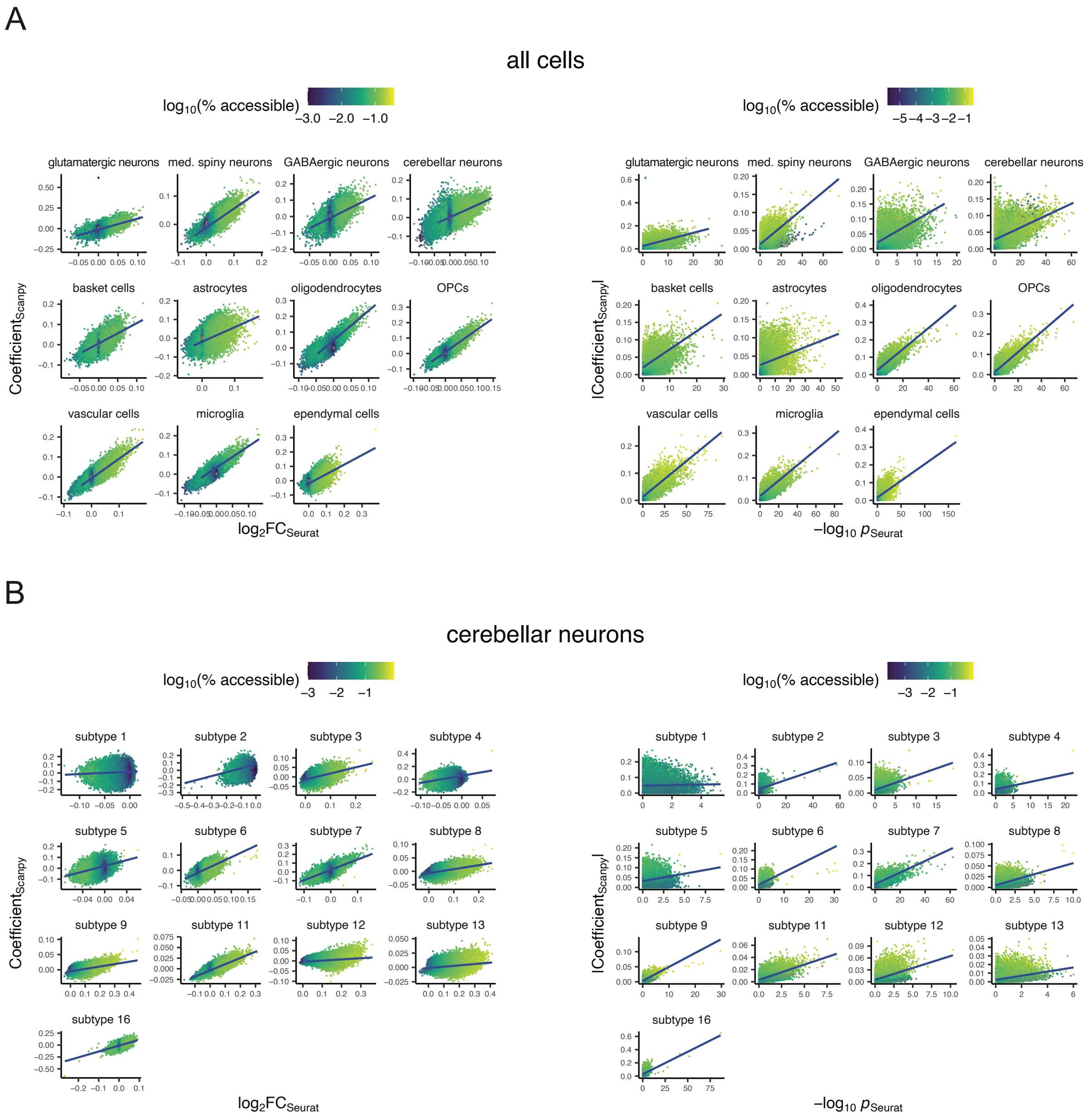

Figure S18. Validation of marker peaks. Due to the large size of the data, the relatively computationally tractable regularized logistic regression method implemented in Scanpy was used to calculate differential accessibility across peaks. The regularized logistic regression, however, is unable to calculate P values or to control for covariates such as UMI counts. We therefore subsampled our dataset (1,000 cells per cell class or subtype) and repeated the analysis using the Seurat implementation (‘FindMarkers‘) of logistic regression, which allowed us to control for UMI counts and to calculate P values. We confirmed that results were similar, both at the **A**, class, and **B**, subtype levels (a representative subtype-level analysis is shown here for the cerebellar neuron data partition).

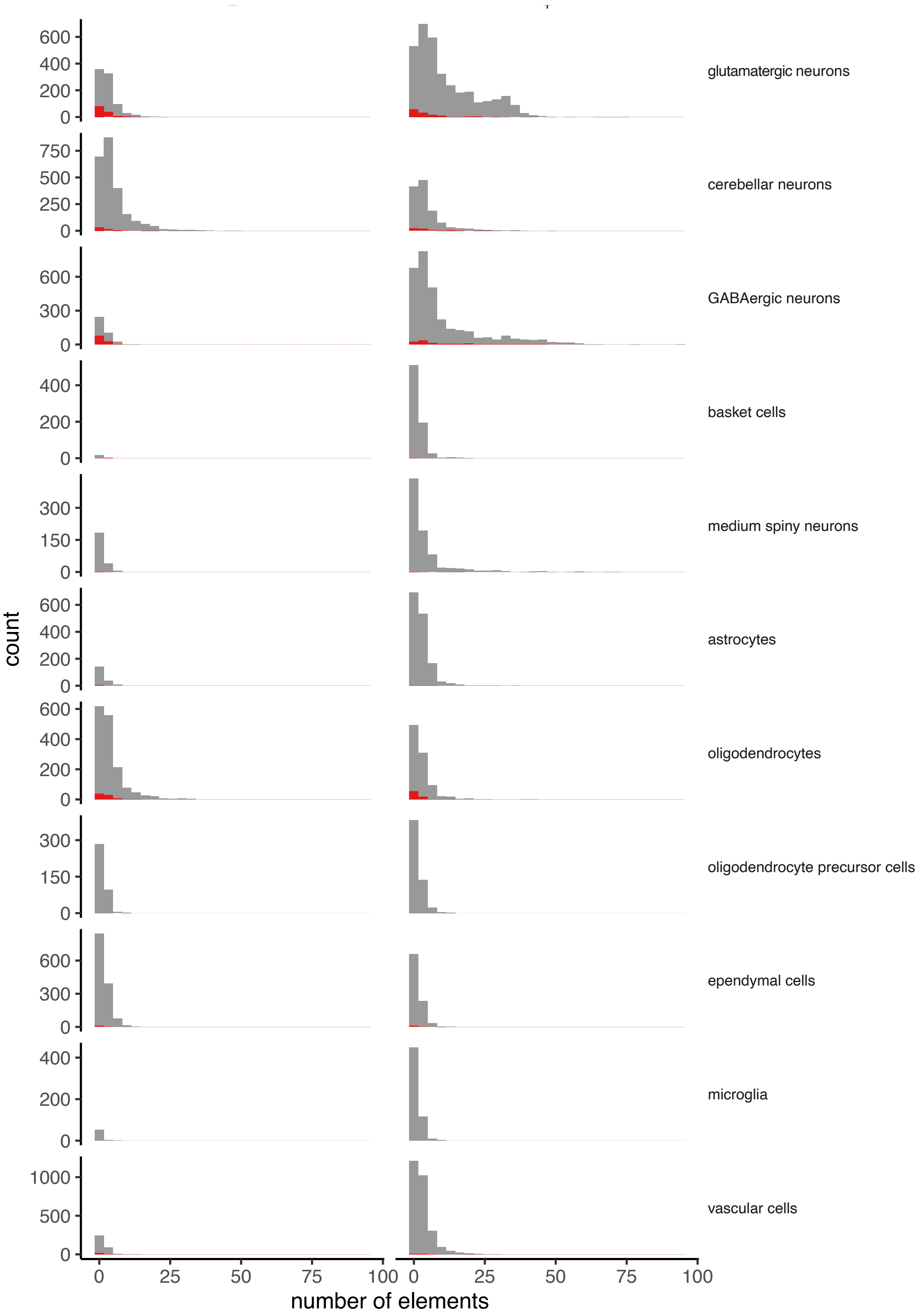

Figure S19. Histograms showing the number of cCREs (peaks) identified as interacting with a gene for each of 11 cell classes. Interactions are grouped separately into positive and negative interactions. Genes having both positively and negatively associated cCREs are shaded in red.

Supplementary Tables

Table captions listed here. Full tables are in the supplementary Excel file.

Table S1. List of brain regions sampled in this study. Cell counts for both snRNA-seq and snATAC-seq are final counts after all quality control filters have been applied.

Table S2. Sample metadata for this study. Animal IDs and social group IDs follow the identifiers assigned by the Caribbean Primate Research Center (CPRC) for the Cayo Santiago macaque colony. Brain regions follow the abbreviations in **table S1**. Hemispheres with `<NA>` values indicate that samples are from midline structures and were sampled as a single sample from either or both hemisphere(s). Library IDs and batch IDs for both sci-RNA-seq3 and sci-ATAC-seq3 are internal identifiers in use by the Brotman Baty Institute Advanced Technology (BAT) Laboratory. Cell counts for both snRNA-seq and snATAC-seq are final counts after all quality control filters have been applied.

Table S3. Cell class statistics and marker genes. Counts for each cell class are included for both the snRNA-seq and snATAC-seq datasets. The comma-separated list of top 10 marker genes for each cell class is included, ranked in descending order by regularized logistic regression coefficient.

Table S4. Oncobox pathway analysis results. Pathway activity levels (PALs) were calculated for each cell class, and pathways with the 50 highest and 50 lowest average PALs are listed, after first filtering out results from duplicated pathways found in multiple database versions in OncoboxPD.

Table S5. Gene Ontology enrichment results. Enrichment analyses were run separately for each test direction, cell class, and GO namespace. GO classes are considered significantly enriched where $P_{adj} < 0.01$.

Table S6. Cell-class compositions across sampled brain regions in snRNA-seq dataset. Relative proportions are for cell classes (rows) found in each brain region (columns) in the snRNA-seq dataset.

Table S7. Cell subtype statistics and marker genes. Counts for each cell subtype are included for both the snRNA-seq and snATAC-seq datasets. The comma-separated list of top 5 marker genes for each cell subtype is included, ranked in descending order by regularized logistic regression coefficient (testing expression of each subtype compared to expression in other subtypes within a given cell-class partition). In some cases, cell subtypes were annotated based on non-negative least-squares (NNLS) regression comparisons with external datasets. When applicable, cell-type labels and DOIs are included for NNLS matches.

Table S8. Cell subcluster statistics and marker genes. The comma-separated list of top 5 marker genes for each cell subtype is included, ranked in descending order by *t*-test score (testing expression of each subcluster compared to the union set of neighboring cells).

Table S9. Cell-subtype compositions across sampled brain regions in snRNA-seq dataset. Relative proportions are for cell subtypes (rows) found in each brain region (columns) in the snRNA-seq dataset.

Table S10. Genes associated with lochNESS at the region subclass level in the astrocytes. Generalized linear models were fit to each variable gene in the astrocytes. For each gene and region subclass, an estimate and adjusted *p*-value is calculated based on the model. Gene and region subclass pairs with significant non-zero estimates ($P_{adj} < 0.05$) are included in the table.

Table S11. Enrichment of transcription factor binding motifs among cell-class-specific peaks. Enrichments were performed on peaks that were called separately for each cell class and that showed no genomic overlap with peaks called using other cell classes. Enrichment was performed using monaLisa on JASPAR transcription factor (TF) binding profiles, using the rhesus macaque genome as the background. TF binding motifs with $P_{adj} < 0.05$ for a given cell class are included in this table.

Table S12. Enrichment of transcription factor binding motifs among cell-subtype differentially accessible peaks. Enrichments were on peaks that were called separately for each cell class and that were classified as differentially accessible marker peaks, defined as being in the top percentile (1%) of peaks (ranked by regularized logistic regression coefficient) with regularized logistic regression coefficient > 0. Enrichment was performed using monaLisa on JASPAR transcription factor (TF) binding profiles, using the rhesus macaque genome as the background. The top 20 TF binding motifs with $P_{adj} < 0.05$ (ranked by P_{adj}) for a given cell subtype are included in this table.

Table S13. Enrichment of disease-associated genes among cell-subtype marker genes. Enrichment of disease-associated genes was performed using disease association from the DISEASES database. Enrichment of disease associations among the top 100 marker genes (*t*-test $P_{adj} < 0.05$; ranked by regularized logistic regression coefficient) for each cell subtype were performed using Fisher's exact test.

Table S14. Correlation between transcription factor expression and motif enrichment. A total of 369 JASPAR TFs with a corresponding gene in the snRNA-seq dataset were included in the analysis. The mean expression and mean motif enrichment of each TF were calculated for each cell type and region class. Correlation between gene expression and motif enrichment across cell type and region classes was quantified with the Pearson's correlation coefficient.

Table S15. Candidate *cis*-regulatory interactions between peaks and genes (global). Regulatory inference results using peaks called on the global (all cells combined) dataset. Peak-gene pairs were considered significant if $P_{adj} < 0.05$ for both regulatory inference methods (GLUE regulatory inference and metacell-based logistic regression). Because of the large number of identified candidate regulatory interactions, only results with $P_{adj} < 0.005$ for both statistics are included in this table.

Table S16. Candidate *cis*-regulatory interactions between peaks and genes (cell classes). Regulatory inference results using peaks called on the dataset partitioned by cell class. Peak-gene pairs were considered significant if $P_{adj} < 0.05$ for both regulatory inference methods (GLUE regulatory inference and metacell-based logistic regression). Because of the large number of identified candidate regulatory interactions across cell classes, only results with $P_{adj} < 0.001$ for both statistics are included in this table.

Table S17. Enrichment results (Fisher's exact tests) for evolutionarily salient gene sets (listed in the "Human genomic region set" column: DA_{hc} = differentially accessibly in human vs. chimpanzee organoids; HAR = human accelerated regions; HAQER = human ancestor quickly evolved regions) among cCREs identified in the current study (versus all peaks), cCREs identified as differentially accessible across cell classes (versus all cCREs, called globally), or cCREs identified as cell class specific (versus all cCREs called in each cell class) (target and background sets are listed in "Macaque genomic region set" and "Macaque genomic region background" columns, respectively).

Table S18. List of phenotypes included in linkage disequilibrium score (LDSC) enrichment analysis. Genome-wide association study (GWAS) citations and digital object identifiers (DOIs) are included.

Table S19. Linkage disequilibrium score (LDSC) enrichment results. LDSC enrichment was performed on peaks called separately for each cell class. All results with $P_{adj} < 0.05$ are included in this table.
